# Thermoelectric Array Sensors with Selective Combustion Catalysts for Breath Gas Monitoring

**DOI:** 10.3390/s18051579

**Published:** 2018-05-16

**Authors:** Woosuck Shin, Tomoyo Goto, Daisuke Nagai, Toshio Itoh, Akihiro Tsuruta, Takafumi Akamatsu, Kazuo Sato

**Affiliations:** 1 National Institute of Advanced Industrial Science and Technology (AIST), Shimo-Shidami, Moriyama-ku, Nagoya 463-8560, Japan; d-nagai@aist.go.jp (D.N.); itoh-toshio@aist.go.jp (T.I.); a.tsuruta@aist.go.jp (A.T.); t-akamatsu@aist.go.jp (T.A); 2 The Institute of Scientific and Industrial Research, Osaka University, Ibaraki 567-0047, Japan; goto@sanken.osaka-u.ac.jp; 3 Department of Mechanical Engineering, Aichi Institute of Technology, Toyota 470-0392, Japan; sato@aitech.ac.jp

**Keywords:** thermoelectric device, array sensor, H_2_, CO, CH_4_, combustion catalyst, micro-electromechanical systems (MEMS)

## Abstract

Inflammable breath gases such as H_2_ and CH_4_ are used as bio markers for monitoring the condition of the colon. However, their typical concentrations of below 100 ppm pose sensitivity and selectivity challenges to current gas sensing systems without the use of chromatography. We fabricated a compact, gas-selective thermoelectric array sensor (TAS) that uses micro-machined sensor devices with three different combustion catalysts to detect gases such as H_2_, CO, and CH_4_ in breath. Using Pt/Pt-W thin-film micro-heater meanders, Pd/Al_2_O_3_, Pt,Pd,Au/Co_3_O_4_, and Pt/Al_2_O_3_ catalysts were heated to 320, 200, and 125 °C, respectively, and the gas sensing performances of the TAS for each gas and for a model breath gas mixture of 100 ppm H_2_, 25 ppm CO, 50 ppm CH_4_, and 199 ppm CO_2_ in air were investigated. Owing to its high catalyst temperature, the Pd/Al_2_O_3_ catalyst burned all three gases, while the Pt,Pd,Au/Co_3_O_4_ burned CO and H_2_ and the Pt/Al_2_O_3_ burned H_2_ selectively. To calibrate the gas concentration of the mixture gas without the use of a gas separation tool, linear discriminant analysis was applied to measure the sensing performance of TAS. To enhance the gas selectivity against H_2_, a double catalyst structure was integrated into the TAS sensor.

## 1. Introduction

Human breath is a highly complex mixture of more than 100 types of gases, including NO, CO_2_, CO, NH_4_, CH_4_, H_2_, and various volatile organic components (VOCs), many of which can provide useful information in the monitoring of the human health condition [1]. H_2_ and CH_4_ are generated in the human body by the fermentative reaction of bacteria, enabling the evaluation of the digestive and absorptive functions in the intestine based on measurements of these gases in exhaled breath [2]. CO is exhaled by smokers and has been reported as a marker gas for various diseases [1,3]. 

The techniques developed for the detection and measurement of breath gases can be generally categorized into two major groups: (1) conventional analyzers that use gas chromatography (GC) coupled with mass spectroscopy (MS) or flame ionized detection (FID) [4], recently developed ion mobility spectrometry (IMS) [5] and proton-transfer-reaction (PTR) [6] technologies, and rather simple systems comprising GCs coupled to metal oxide (MOX) sensors; and (2) e-Nose systems, which are commonly constructed as single [7] or array sensors [8]. Although the development of e-Nose systems has been steady over the past few decades, multi-gas sensors for breath gas detection have yet to emerge. Two major stumbling blocks have been the ability to detect specific gases selectively using a single sensing element and the ability to detect gas precisely in humid conditions. 

Low-cost devices for breath analysis without the use of gas chromatography require the use of highly selective and sensitive sensors. Previous studies reported the development of various gas sensors, including semiconducting MOX sensor arrays [9] and micro-hotplate-based gas sensor arrays [10,11]. For the specific analysis of CO and CH_4_ gas mixtures, a micromachined gas sensor based on a catalytic thick film/SnO_2_ thin-film bilayer and a thin-film heater has been developed [12]. Recently, a thermoelectric gas sensor (TGS) based on a synergetic combination of catalytic combustion and thermoelectric conversion has been developed for the detection of H_2_ [13]. Using TGS-based techniques, a gas-flow-type prototype for the selective monitoring of H_2_ in breath gas has been developed and tested [14] and an integrated multi-TGS sensor comprising three thermoelectric array devices with ceramic catalysts for the combustion of CH_4_, CO, and H_2_ has been developed and its performance in detecting these gases at 5000 ppm has been preliminarily tested [15]. Although the detection of the CO and CH_4_ ppm levels was not easy in these studies, the authors have subsequently succeeded in measuring both gases down to 1 ppm [16,17,18]. However, measuring the ppm levels of gas using a single micro-chip-type device remains challenging, with many technical, drift, and process problems remaining, as the extreme fragility of the sensor membranes leads to a low process yield in developing multi-sensor chips with four clean membranes. 

In this study, we improved the sensitivity of a thermoelectric array gas sensor (TAS) by applying the working principle of a TGS with different combustion catalysts for the simultaneous detection of CO, H_2,_ and CH_4_ without gas separation to low-concentration gases at several ppm levels. We investigated the performance of this sensor in an environment of simulated breath gases including water and CO_2_. Our results suggest that the proposed improved TAS is a promising candidate for application as a simple, low-cost breath gas analyzer. 

## 2. Experimental

### 2.1. Integration of Catalyst Combustor onto Array Sensor

**Figure 1:**
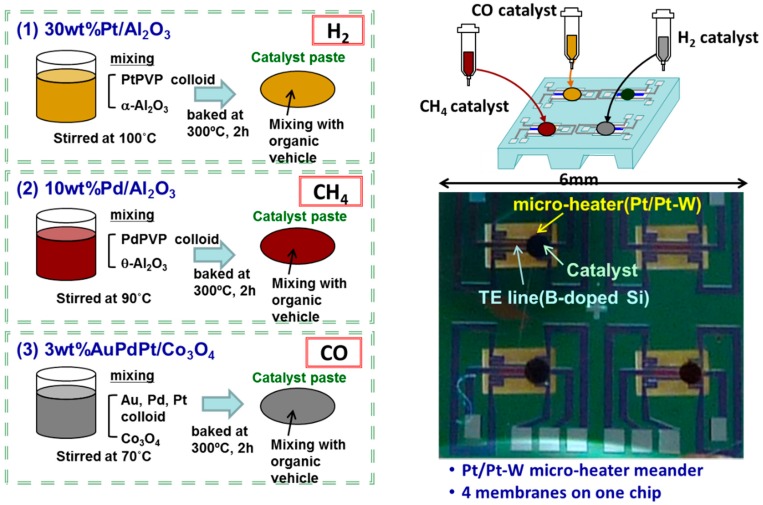
Catalyst combustors deposited onto Pt/Al_2_O_3_, Pd/Al_2_O_3_, and Au/Co_3_O_4_ array sensor devices for the gas detection of H_2_, CH_4_, and CO, respectively.

Figure 1 shows the catalyst deposition process and a photo of the proposed TAS device, which comprises a 6 mm × 6 mm chip containing four thermoelectric device sensors. The sensors are fabricated in a p-type B-doped SiGe pattern and a Pt/Pt-W multilayer micro-heater meander pattern and incorporate combustion catalysts integrated onto a Si_3_N_4_-SiO_2_ multilayer membrane on a double-side polished Si substrate. The B-doped SiGe thin film is prepared using a helicon sputtering method (i-sputter, ULVAC, Inc., Kanagawa, Japan). The thin micro-heater meander films are multi-layer sputter depositions of a 10-nm-thick Ta adhesion layer, a 200-nm-thick Pt layer, and a 10-nm-thick Pt-W layer, and are patterned using a lift-off process. The reverse side of the Si substrate is etched out using an aqueous KOH solution to prepare the membrane structure. Details on the processing and patterning of the SiGe thin film were previously reported in [11]. 

Pt/Al_2_O_3_, Pt,Pd,Au/Co_3_O_4_, and Pd/Al_2_O_3_ combustion catalysts with metal contents of 30, 3, and 10 wt %, respectively, are used for the detection of H_2_, CO, and CH_4_, respectively. The Pt/Al_2_O_3_ and Pd/Al_2_O_3_ catalysts are prepared by mixing an aqueous solution dispersed with a colloid of noble metal particles (Tanaka Kikinzoku Kogyo K.K.; mercaptosuccinic acid, with metal content 4 wt % and average particle diameter 3 nm) and a commercial alumina powder (Taimei Chemicals Co., Ltd., Tokyo, Japan; average particle diameter 100 nm). The Pt,Pd,Au/Co_3_O_4_ catalyst is prepared by mixing an aqueous solution dispersed with the above colloid, as well as a colloid of Au metal particles (Tanaka Kikinzoku Kogyo K.K.; mercaptosuccinic acid, Au content 2 wt %, average particle diameter 3 nm) and a commercial cobalt oxide (Co_3_O_4_) powder (Aldrich; average particle diameter 20–30 nm). The mixture solution is stirred at a temperature of 100, 90, and 70 °C, respectively, until the water evaporates and the solid residue is baked again in air at 300 °C for 2 h to obtain powder catalysts, which are mixed with an organic vehicle (a mixture of terpineol and ethyl cellulose) to form ceramic pastes. The pastes are integrated onto the thin membrane of the TAS using an air dispenser (Musashi Engineering Inc., Tokyo, Japan), and the TAS is then baked in air at 300 °C for 2 h. The size of the catalyst is constrained to a diameter of 0.4 mm, or about 61% of the diameter of a single sensor [13]. After package dicing, the catalysts are deposited onto the TAS device and wire-bonded onto a ceramic package comprising 28 electrode pads (Kyocera Co., Kyoto, Japan, Ceramic Packages for MEMS Sensors). 

### 2.2. Sensor Response Test

The gas response performance of the device was investigated using a gas-flow-type test chamber. The voltage signal from the TAS was recorded by alternately flowing a dry target gas mixture and dry air into the test chamber at a flow rate of 200 mL/min. Performance testing of the TAS was carried out using two types of gas mixture: (1) single gas mixtures of H_2_, CO, or CH_4_ in air; and (2) multiple gas mixtures of H_2_ + CO + CH_4_ in air. The single gases were mixed at 0–100 ppm, while the multiple gases were mixed in the ratio H_2_:100/CO:25/CH_4_:50/CO_2_:199 (ppm). The composition ratio of the test gas is determined to simulate the maximum concentration of three inflammable gases in breath, and the reference gas of the CO_2_ level is fitted to the H_2_ concentration. Details on the gas concentrations in breath has been reported previously [2], which is one hundred times lower than the concentration found in the human colon [19]. 

To measure the gas combustion-induced catalyst temperature change, the surface temperature of the catalyst was monitored using an IR camera that had previously been calibrated using thermocouples. In contrast to our previous experiments, it was difficult to read temperature change using the IR camera, which reflected the differences in concentrations: in the previous experiments, concentrations of hydrogen gas in air as high as 1.0% were measured, leading to catalyst temperature increases of 40 °C; by contrast, the current experiments involved temperature changes as low as 0.005 °C for hydrogen concentrations of 1 ppm.

## 3. Results and Discussion

### 3.1. Combustion Performance of Pt/Al_2_O_3_, Pt,Pd,Au/Co_3_O_4_, and Pd/Al_2_O_3_ Catalysts

The combustion performance of the Pt/Al_2_O_3_, Pt,Pd,Au/Co_3_O_4_, and Pd/Al_2_O_3_ catalysts can be estimated based on the voltage signal of the TAS (ΔV), which is linearly proportional to the increase in the temperature (ΔT) as a result of the gas combustion on the catalyst:

ΔV = αΔT

where α is the Seebeck coefficient of a thermoelectric film of boron-doped Si_0.8_Ge_0.2_.

Table 1 lists the voltage signals, ΔV, produced by the respective TAS catalysts for three inflammable single gases at concentrations of 100 ppm. The sensor operational temperatures—that is, the catalyst temperatures at which the best performances were produced in our previous study [16]—are increased to 150 °C for H_2_ and decreased to 200 and 320 °C, respectively, for CO and CH_4_. Figure S1 in the Supplementary Material shows the typical response curves (in terms of ΔV with respect to time) of the Pd/Al_2_O_3_-catalyst TAS at various gas concentrations of CH_4_ in air with the air flow switched to the gas mixture flow at an elapsed time of 100 s and then switched back to air at 200 s.

**Figure 2:**
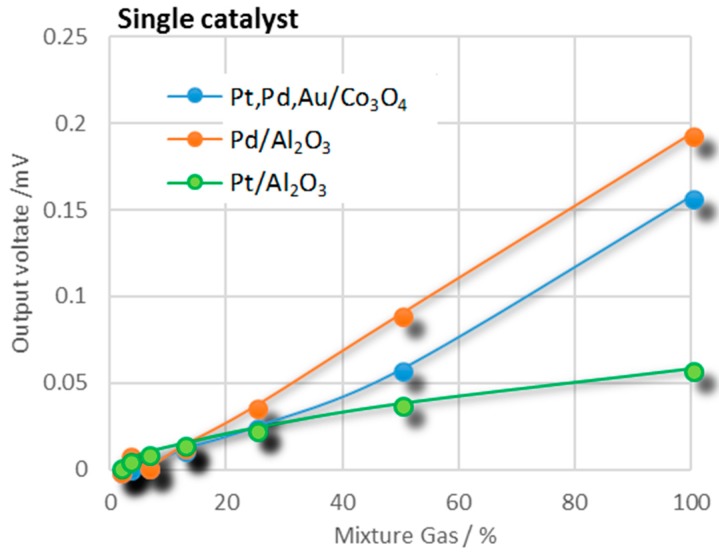
Gas responses or calibration curves of three TAS catalyst sensors for mixture gas with relative component concentrations of H_2_ 100 ppm, CO 25 ppm, CH_4_ 50 ppm, and CO_2_ 199 ppm in air. The *x*-axis represents the concentration of the mixture gas diluted by air.

Figure 2 shows the detection performances of the Pt/Al_2_O_3_, Pt,Pd,Au/Co_3_O_4_, and Pd/Al_2_O_3_-catalyst TASs for a H_2_:100 ppm/CO:25 ppm/CH_4_:50 ppm/CO_2_:199 ppm gas mixture at various concentrations in air. 

From Table 1, the Pt/Al_2_O_3_ catalyst TAS had a combustion performance of ΔV = 0.036 mV for 100 ppm H_2_ at a catalyst temperature of 125 °C. At this temperature, CO was also burned by this catalyst with a ΔV of 0.016 mV for 100 ppm CO. However, the catalyst did not burn CH_4_. From Figure 2, it is seen that the Pt/Al_2_O_3_ catalyst burned the CO + H_2_ + CH_4_ gas mixture more effectively than it burned any of the gases separately. At concentrations above approximately 50%, the gas mixture ΔV exceeded the sum of the values (0.05 mV) of the individual gases at 125 °C, with the ΔV value for the 25 ppm CO in the gas mixture approximately corresponding to that achieved by the 100 ppm CO single gas.

In our previous studies of Pt/Al_2_O_3_ catalysts on micro devices, we found that CO adsorption on the catalyst surface prohibited the combustion of H_2_ at lower temperatures [18], making it necessary to increase the catalyst temperature to burn out the adsorbed CO to activate the catalyst surface. This enhanced combustion of CO could be explained in terms of activation with coexisting H_2_, and these results were used to obtain operating temperatures of 320, 200, and 125 °C, respectively, for Pd/Al_2_O_3_, Pt,Pd,Au/Co_3_O_4_, and Pt/Al_2_O_3_-catalyst TAS devices. The Pt,Pd,Au/Co_3_O_4_-catalyst TAS produced a ΔV of 0.53 mV in burning 100 ppm CO at a catalyst temperature of 200 °C and 0.56 mV in burning 100 ppm H_2_, but could not burn CH_4_. The ΔV = 0.158 mV obtained by burning the H_2_ + CO + CH_4_ gas mixture in air at 200 °C was greater than the sum of the ΔV values (0.0825 mV) obtained by burning the single gases separately at the same temperature. 

The Pd/Al_2_O_3_ catalyst burned all three gases at a catalyst temperature of 320 °C, producing nearly identical values of ΔV for H_2_ and CO, but a value approximately twice that for CH_4_. At catalyst temperatures above 350 °C, the combustion of CH_4_ by the catalyst increased to even higher values relative to that with CO. The CH_4_ selectivity, S_CO/CH4_ = ΔV _CO_/ΔV _CH4_, at a catalyst temperature of 360 °C, was 0.5. At a catalyst temperature of 320 °C, the TAS sensor was able to detect CO and CH_4_ down to a 1-ppm concentration level. However, several technical problems were encountered: the base line of the sensor voltage signal was unstable and drifted, and the linearity of V with respect to gas concentration became worse. 

### 3.2. Double Catalyst to Enhance TAS Gas Selectivity

As demonstrated in Table 1 and Figure 2, linear discriminant analysis (LDA) could be applied in assessing combustion performance. However, the gas selectivity of TAS is low, particularly for CO and CH_4_, and we therefore attempted to control the selectivity of these gases using a “double catalyst structure” [8,19] to adjust the balance of the combustion heats of the catalysts deposited onto the thermoelectric film in the micro-TGS devices to obtain further improvements in TAS selectivity. 

**Figure 3:**
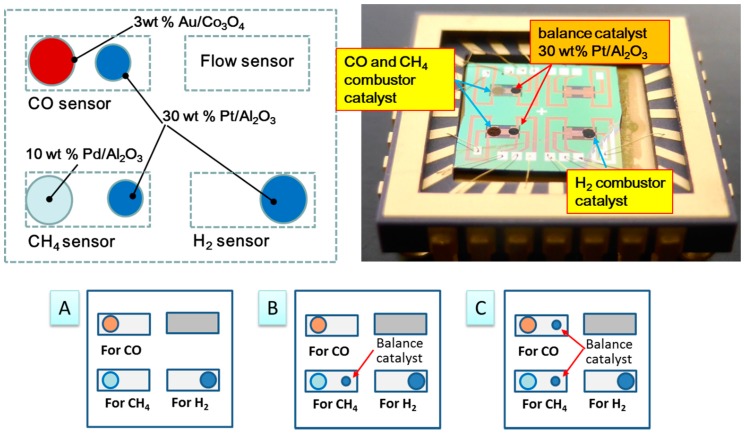
Additive deposition of 30 wt % Pt/α-Al_2_O_3_ catalyst, which combusts H_2_ gas to enhance gas selectivity. (**A**) Schematic of C-type TAS; (**B**) Following package dicing, catalysts are deposited onto the TAS device and wire-bonded onto a ceramic package; (**C**) Positioning of additive 30 wt % Pt/α-Al_2_O_3_ catalyst for type-B and -C TASs.

As shown in Figure 3, single and double 30 wt % Pt/α-Al_2_O_3_ catalysts were deposited onto the cold ends of separate thermoelectric patterns to produce two new TAS types (B and C, respectively, with type-A indicating the original TAS configuration). Here, the hot side indicates the location of the combustion catalyst, while the counter or cold side refers to the position of the additional catalyst on the membrane. 

As an example of the effects of the double catalyst process, the deposition of the Pt,Pd,Au/Co_3_O_4_ catalyst (at point A) should allow for the oxidization of both H_2_ and CO. Depositing Pt/α-Al_2_O_3_, which is a combustion catalyst for H_2_ [13], onto the other side of membrane (point B) should reduce the temperature difference between points A and B owing to the combustion heat of the Pt/α-Al_2_O_3_. As a result, introducing a mixture of H_2_ and CO into this calorimetric-TGS device will inhibit the sensor response of the Pt,Pd,Au/Co_3_O_4_ catalyst to H_2_.

**Figure 4:**
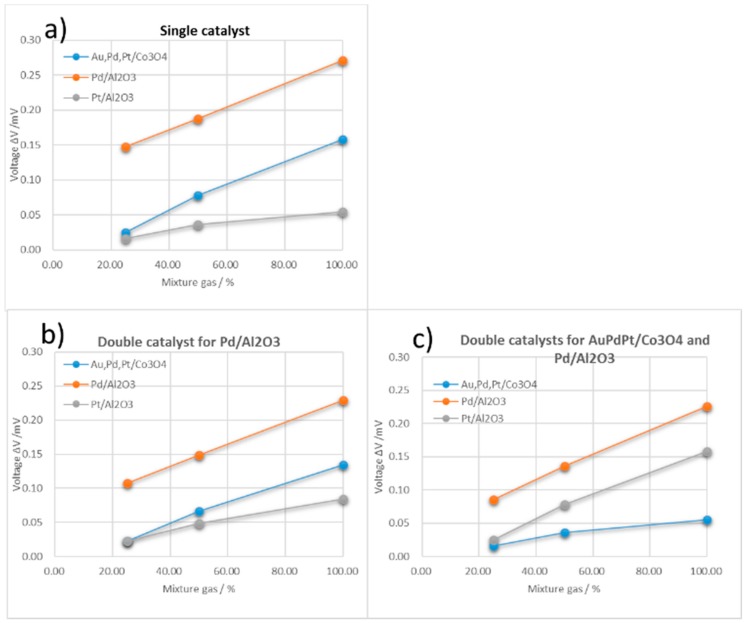
Gas responses or calibration curves of TASs of types (**a**–**c**) for a mixture gas with relative component concentrations of H_2_ 100 ppm, CO 25 ppm, CH_4_ 50 ppm, and CO_2_ 199 ppm in air. The *x*-axis represents the concentration of the air-diluted mixture gas.

The response ΔV values of A-, B-, and C-type TASs were measured for single and mixture gases. Because of the CO absorption on the Pt/Al_2_O_3_ catalyst, the driving temperature of the Pt/Al_2_O_3_ catalyst was increased to 150 °C to enable burning the CO. Figure 4 shows the responses of the three TAS types for a H_2_ 100 ppm, CO 25 ppm, CH_4_ 50 ppm, and CO_2_ 199 ppm gas mixture in air, which can be used for calibration curves. The responses for single and mixture gases are listed in Table 2. 

Figure 4a shows the mixture-gas response for the type-A TAS, which as noted above, has the same structure as the TAS discussed in the preceding sections. Unsurprisingly, the reaction is very similar to that shown in Figure 2, although the ΔV values produced by the methane and CO sensors are higher; this difference can be attributed to the catalyst size difference between the respective devices, with the larger ΔV representing a response to a larger signal. 

Figure 4b shows the response of the type-B TAS, which has a double catalyst in its CH_4_ sensor, to the gas mixture. Although the response is essentially the same as that of the type-A TAS, the methane sensor in the former has a lower ΔV than the sensor in the former. This reduced ΔV can be attributed to the combustion of H_2_ and CO gas on the Pt/Al_2_O_3_ catalyst on the type-B methane catalyst. As seen in Figure 4c, this double catalyst effect becomes more prominent in the CO sensor of the type-C TAS with double catalysts of Pt,Pd,Au/Co_3_O_4_ and Pt/Al_2_O_3_. While the ΔV for the mixture gas of the CO catalyst of the type-A TAS is equal to the sum of the ΔV values of each single gas, the mixture gas response of the type-C catalyst is close to that for 25 ppm CO in air, i.e., the catalyst is CO selective. The CH_4_ sensor of the type-C TAS produces similar response levels, demonstrating the effect of the double catalyst. 

While all of the methane sensor responses increase linearly with gas concentration (R^2^ > 0.998), the linearities of the CO (R^2^ > 0.995) and H_2_ sensors (R^2^ >0.956) are lower and the increases in their ΔV values decline in the lower ppm ranges. Based on this linear performance of the sensors, LDA analysis was applied to the sensing performance factors shown in Table 2 and Figure 4 to evaluate and distinguish the composition of the mixture gas. 

**Figure 5:**
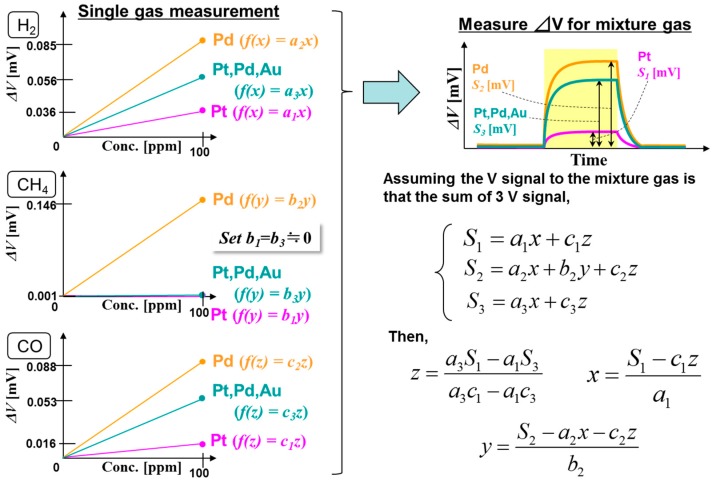
Schematic of the linear analysis method used in this work.

The method for using multivariate analysis and calibration models built individually for each type of sensor based on the previously identified single-gas responses is illustrated in Figure 5, where Sn represents the parameters extracted from the response signals of the respective sensors (*n* = 1, 2, 3) and a, b, c are coefficients indicating the respective gas components. The sensor responses are normalized to that for a 100-ppm single gas in air—for example, S_1_ = a_1_x + b_1_y + c_1_z. The coefficients of the gas composition equation can then be calculated under a simple assumption that the mixed gas ΔV is the sum of the single gas response signals. As the immunity of TGS to water vapor has been validated many times [13,14] and demonstrated in the hospital [20], the effect of water vapor can be ignored. The results for the three TAS types for different mixture gas compositions are listed in Table 3. The validity of the proposed method is validated by the closeness of these coefficients to the original mixture gas compositions.

Using data on the linear relation of V to gas concentration, calibration can be applied to adjust the gas concentration, for example, from 86 to 50 ppm CH_4_ for the type-A TAS. This can provide simple gas composition estimates if the sensor response is stable throughout the calibration process and in actual use. The effect of H_2_ combustion as a result of the double catalyst effect can be further used to modify the responses of the types-B and -C TASs, as shown in Figure 5. However, the linear equation analysis returns a negative CO gas composition for the type-C TAS, making it difficult to adjust the gas composition in this case, and reflecting the need of further modification and improvement of the double catalyst method for the Pt,Pd,Au/Co_3_O_4_ and Pt/α-Al_2_O_3_ CO sensor. The type-B TAS estimates relatively low H_2_ concentrations, which results in relatively high CH_4_ and CO responses. These results suggest that the type-A TAS is most suitable for discriminating separate gas compositions using linear analysis, while the double catalyst types-B and -C TASs require further modification in terms of, for example, catalyst size or temperature, even though both sensors have enhanced selectivity against H_2_. 

We are currently developing a breath detection system based on the use of a TAS structure for which the linear modeling results of this study will be potentially useful in calibrating the sensors in operating mode. As the sensor response changes as a result of altered installation packages or gas flow, the linear calibration method demonstrated in this study can be used to explore, test, and calibrate the performance of new array sensor packages with respect to specific operating modes. However, such calibration challenges can also be overcome by combining sensors into an array (e.g., E-nose) in which differences in selectivity are exploited through statistical analysis. Further work needs to be carried out, the calibration for the accuracy of the gas concentration with the estimation error analysis is necessary, and the cross-validation with human breath, including the interference of gas mixtures of very low concentration analytes, should be tested carefully, for instance, acetone, which is of several ppm in breath gas of diabetes.

## 4. Conclusions

TASs based on Pd/Al_2_O_3_, Pt,Pd,Au/Co_3_O_4_, and Pt/Al_2_O_3_ combustion catalysts were developed and assessed in terms of selective breath gas detection performance for the inflammable gases H_2_, CO, and CH_4_ at a variety of concentrations. Sensors of each catalyst type were fabricated as micro-machined membranes and heated using Pt/Pt-W thin film heaters to 320 °C (Pd/Al_2_O_3_), 200 °C (Pt,Pd,Au/Co_3_O_4_), and 125 °C (Pt/Al_2_O_3_), with the gas concentrations detected via the thermoelectric conversion of combustion heat to a Seebeck voltage in linear proportion to the gas concentration. 

The TAS responses for the single composition gases were evaluated separately and the responses for mixture gases were tested to confirm that the sensors could detect the concentrations of the three gases selectively and linearly. To enhance the limited gas selectivity against H_2,_ a double catalyst structure was integrated into the sensor membrane, which was shown to be effective in enhancing the sensor performance and enabling a more favorable sensor design.

Based on this linearization of combined measurement results, it is possible to estimate the concentrations of individual gases using a TAS, which could facilitate the simpler screening of lung cancer patients and monitoring of breath gas concentrations. In future research, we will seek to improve the sensitivity of the proposed thermoelectric multi gas sensor to gases at concentrations at the several ppm level and to investigate the performance of this enhanced sensor in measuring simulated real-environment gases such as water and CO_2_. We believe that the sensor developed in this study represents a promising candidate for use in a simple, low-cost breath gas analyzer.

## Figures and Tables

**Table 1:** Combustion performance of sensors with different catalyst materials separately exposed to single inflammable gases H_2_, CH_4_, and CO at air concentrations of 100 ppm. The sensors were heated separately to avoid interference.

Catalyst Material	Catalyst	ΔV for Single Gas at 100 ppm (mV)		
	Temp. (°C)	H_2_	CH_4_	CO
Pt/Al_2_O_3_	125	0.036	0.001	0.016
Pt,Pd,Au/Co_3_O_4_	200	0.056	0.001	0.053
Pd/Al_2_O_3_	320	0.085	0.146	0.088

**Table 2:** Combustion performance of TASs without/with double catalyst structure (A/B and C, respectively) and different catalyst materials for single inflammable gases H_2_, CH_4_, and CO at 200 ppm/air and for gas mixtures.

**Single Catalyst Type A**						
**Catalyst**	**Single Gas 100 ppm**			**Mix Gas (%)**		
	**H_2_**	**CH4**	**CO**	**25**	**50**	**100**
Pt/Al_2_O_3_	0.0386	0.0034	0.0256	0.016	0.036	0.055
Pd/Al_2_O_3_	0.142	0.164	0.082	0.18	0.229	0.33
Pt,Pd,Au/Co_3_O_4_	0.0307	0.12	0.083	0.025	0.078	0.158
**One Double Catalyst Type B**						
**Catalyst**	**Single Gas 100 ppm**			**Mix Gas (%)**		
	**H_2_**	**CH4**	**CO**	**25**	**50**	**100**
Pt/Al_2_O_3_	0.230	0.000	0.033	0.025	0.078	0.158
Double	0.125	0.176	0.027	0.086	0.136	0.226
Pt,Pd,Au/Co_3_O_4_	0.045	0.003	0.055	0.016	0.036	0.055
**Two Double Catalyst Type C**						
**Catalyst**	**Single Gas 100 ppm**			**Mix Gas (%)**		
	**H_2_**	**CH4**	**CO**	**25**	**50**	**100**
Pt/Al_2_O_3_	0.150	−0.030	−0.030	0.023	0.0668	0.134
Double	0.092	0.146	−0.003	0.108	0.149	0.229
Double	0.110	0.120	0.260	0.0232	0.0485	0.152

**Table 3:** Gas concentration for three TAS types by mixture gas composition.

	Type A	Type B	Type C	Mixture Gas Composition
H_2_ conc. (x)	121	62	108	100 ppm
CH_4_ conc. (y)	86	77	89	50 ppm
CO_2_ conc. (z)	21	46	−4	25 ppm

## Supplementary Materials

The following are available online at http://www.mdpi.com/1424-8220/18/5/1579/s1, Figure S1: Gas response curves of the TAS with Pd/Al_2_O_3_ catalysts for the various concentration of CH_4_ in air.
