# Supplementary material for: Thermoelectric Array Sensors with Selective Combustion Catalysts for Breath Gas Monitoring
Source: Sensors (Basel). 2018 May 16;18(5):1579. doi: 10.3390/s18051579 (PMC5982651; doi:10.3390/s18051579)
Supplement: Supplementary file 1 [file sensors-18-01579-s001.pdf]

sensors

MDPI

Supplementary

Thermoelectric Array Sensors with Selective Combustion Catalysts for Breath Gas Monitoring

Woosuck Shin [1,*], Tomoyo Goto [2], Daisuke Nagai [1], Toshio Itoh [1], Akihiro Tsuruta [1], Takafumi Akamatsu [1] and Kazuo Sato [3]

Supplementary

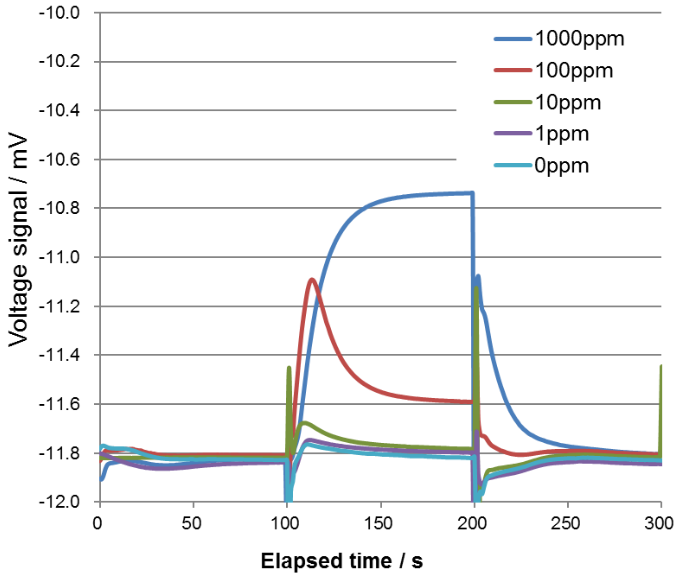

Figure S1. Gas response curves of the TAS with Pd/Al_2O_3 catalysts for the various concentration of CH_4 in air. The change of ΔV from the device, or output voltage signal, V, of TAS for different gas concentrations with respect to time. At elapsed time 100s, the air flow switched to the gas mixture flow, and switched back to air at 200s.

© 2016 by the authors. Submitted for possible open access publication under the terms and conditions of the Creative Commons Attribution (CC-BY) license (http://creativecommons.org/licenses/by/4.0/).
